# Blocking the dimerization of polyglutamine-expanded androgen receptor protects cells from DHT-induced toxicity by increasing AR turnover

**DOI:** 10.1016/j.jbc.2024.107246

**Published:** 2024-03-29

**Authors:** Allison Lisberg, Yuhong Liu, Diane E. Merry

**Affiliations:** Department of Biochemistry and Molecular Biology, Sidney Kimmel Medical College, Thomas Jefferson University, Philadelphia, Pennsylvania, USA

**Keywords:** neurodegeneration, polyglutamine disease, protein folding, protein motifs, androgen receptor, neuromuscular disease, dimerization

## Abstract

Spinal and bulbar muscular atrophy (SBMA) is a neuromuscular degenerative disease caused by a polyglutamine expansion in the androgen receptor (AR). This mutation causes AR to misfold and aggregate, contributing to toxicity in and degeneration of motor neurons and skeletal muscle. There is currently no effective treatment or cure for this disease. The role of an interdomain interaction between the amino- and carboxyl-termini of AR, termed the N/C interaction, has been previously identified as a component of androgen receptor-induced toxicity in cell and mouse models of SBMA. However, the mechanism by which this interaction contributes to disease pathology is unclear. This work seeks to investigate this mechanism by interrogating the role of AR homodimerization- a unique form of the N/C-interaction- in SBMA. We show that, although the AR N/C-interaction is reduced by polyglutamine-expansion, homodimers of 5α-dihydrotestosterone (DHT)-bound AR are increased. Additionally, blocking homodimerization results in decreased AR aggregation and toxicity in cell models. Blocking homodimerization results in the increased degradation of AR, which likely plays a role in the protective effects of this mutation. Overall, this work identifies a novel mechanism in SBMA pathology that may represent a novel target for the development of therapeutics for this disease.

Spinal and bulbar muscular atrophy (SBMA) is an X-linked neurodegenerative disease that is caused by a CAG repeat expansion in the androgen receptor (AR) gene (1). This mutation leads to a polyglutamine (polyQ) tract expansion in the amino terminus of the AR protein and causes the AR to misfold and aggregate, forming nuclear inclusions (2, 3). This misfolding leads to proteotoxicity in lower motor neurons and skeletal muscle, causing progressive skeletal muscle weakness, dysphagia, dysarthria, and eventually morbidity of the patient (4, 5). Toxicity is dependent on AR binding to its ligand, either testosterone or its derivative DHT (6, 7, 8). Additionally, although some patients display signs of loss of AR function in the form of symptoms of androgen insensitivity, it is clear that this is not the primary mechanistic basis of this disease (9). Despite this understanding of some of the molecular events that lead to polyglutamine-expanded AR toxicity, there is currently no cure or widely effective, disease-altering treatment for SBMA, indicating a need for continued investigation into the molecular mechanisms involved in this disease.

The inhibition of AR activity through the use of either AR antagonists or androgen deprivation prevents AR aggregation and toxicity in cell models of SBMA (10, 11, 12) and preserves motor function in mouse models of SBMA (6, 7, 13). However, chemical castration with Leuprorelin has proven effective only in a subset of patients, highlighting the need for further understanding of the pathological mechanisms of SBMA (14). Previous research has shown that preventing the interaction between the amino and carboxyl termini of AR, termed the N/C interaction, may explain the protection offered by AR antagonists, as this interaction is prevented when AR activity is blocked (12, 15). Blocking the N/C-interaction reduces AR aggregation in cell models, prevents cell death in cultured motor neurons, and preserves motor function in mouse models of SBMA, similar to the effects of complete blockage of AR activity (12, 16). The mechanisms behind this protection, however, remain poorly understood, and further research is required to better understand the mechanisms involved to better design treatments that might be able to mimic these effects in patients.

The AR N/C interaction can occur in two places in the AR metabolic cycle. After binding to its ligand, the AR undergoes a conformational change that dissociates the aporeceptor complex, in which inactive AR is held in the cytoplasm and allows for AR import into the nucleus. This conformational change involves binding of the ^23^FQNLF^27^ domain in the N-terminus of AR to the AF2 domain within the AR C-terminal domain (17, 18, 19). This interaction results in the intramolecular form of the N/C interaction and occurs in AR monomers (20). Once imported into the nucleus, the AR forms homodimers and binds to DNA, exerting its function as a transcriptional regulator. In order to carry out this function, AR undergoes another conformational shift, with two AR monomers aligning in an antiparallel configuration and the N/C interaction shifting from an intramolecular to an intermolecular interaction between the two components of the AR homodimer (20, 21). Previous research has shown that this intermolecular interaction occurs primarily within the nucleus (20), although cytoplasmic homodimers have been observed (22). Additionally, blocking this intermolecular interaction has been shown to result in a subsequent increase in intramolecular interaction, although the reverse situation has not been observed (21).

A consideration of the mechanism underlying the protection offered by blocking the N/C interaction may involve the difference between intra- and inter-molecular versions of the N/C interaction. Indeed, although R-bicalutamide, an AR antagonist, inhibits the AR N/C interaction (12, 23, 24), molecular dynamic simulations suggest that R-bicalutamide also causes destabilization of the AR homodimer (25). AR dimers are formed through the antiparallel alignment of three residues in the second zinc finger of the DNA binding domain (A597/S598/T603) which form three hydrogen bonds connecting the dimer partners (26). These residues are not the only ones that play a role in AR dimerization, as an interaction between the ligand-binding domains of the AR has also been described (27, 28, 29, 30, 31) and the N/C-interaction itself contributes to dimerized AR structure and function (32, 33, 34).

In the current study, we sought to elucidate the contribution of the intermolecular N/C interaction in polyglutamine-expanded AR aggregation and toxicity by examining the role of AR homodimerization in SBMA cell models. We show that AR homodimerization is increased in cell models of SBMA expressing polyglutamine-expanded AR. Additionally, blocking homodimerization *via* the mutation of A597/S598 reduced AR aggregation and altered AR localization and stabilization upon ligand binding. Additionally, dimerization-incompetent AR (dimerization mutant or DM AR) offered some protection from toxicity in cultured motor neurons. Our studies further revealed that the effects of blocking dimerization are likely not due to differences in the trafficking of AR but rather to enhanced AR turnover. Taken together, our data suggest that blocking AR dimerization may play a significant role in the protection offered by blocking the AR N/C interaction.

## Results

### The AR N/C-interaction is decreased in SBMA cell models

**Figure 1:**
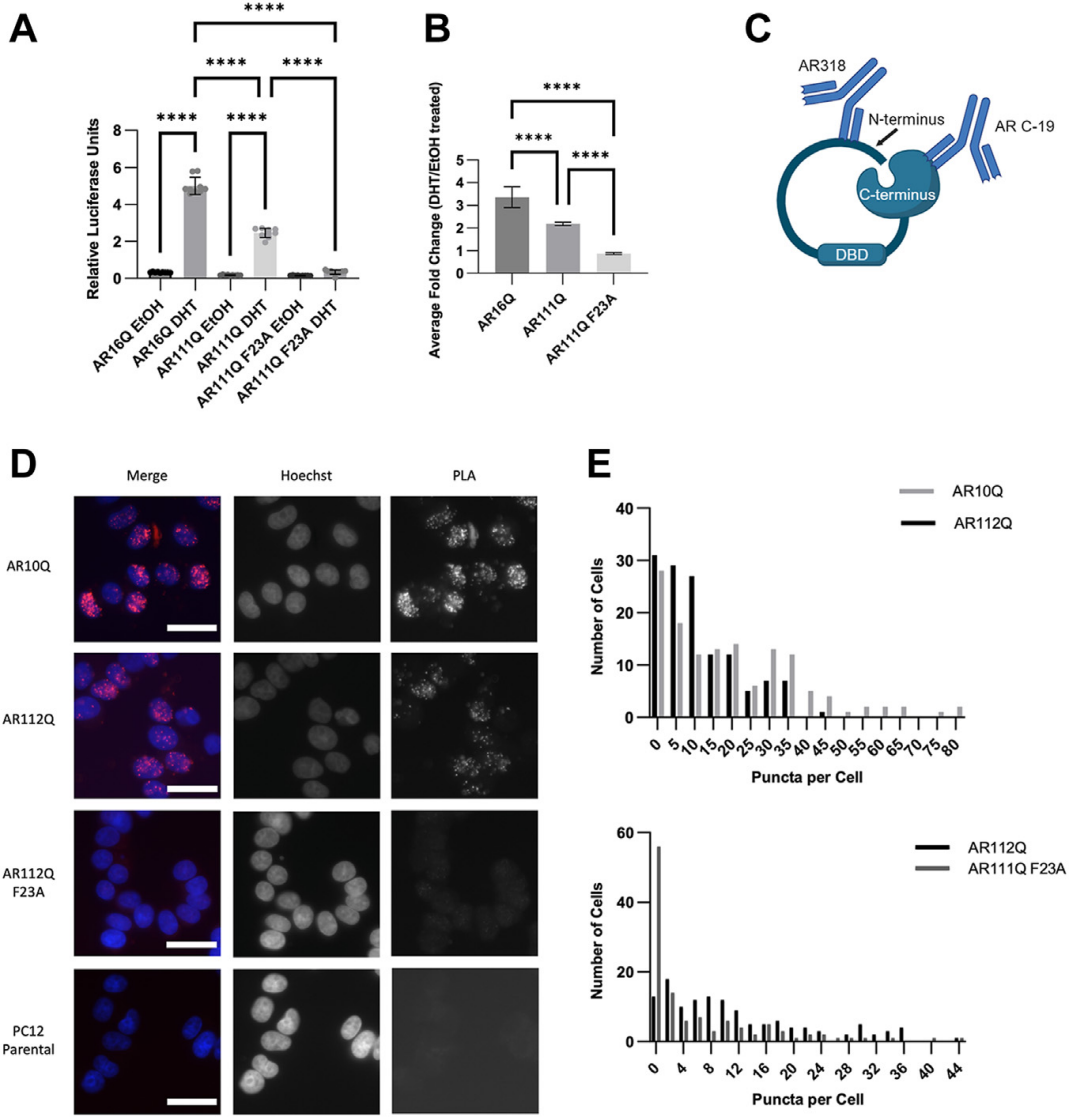
**The AR N/C interaction is decreased by polyglutamine expansion.***A*, the AR N/C interaction was evaluated using mammalian two-hybrid analysis in HEK293T cells. AR111Q N/C-interaction was reduced in the presence of DHT when compared to AR16Q, although not to the level of complete N/C-interaction inhibition. (∗∗∗∗*p* < 0.0001), one-way ANOVA with post hoc Tukey test. *B*, average fold change induction of the N/C interaction in response to DHT, as shown in panel (*A*). *C*, schematic of proximity ligation assay (PLA) used to evaluate the AR N/C interaction. The AR antibodies AR318 and ARC19 were used to probe for the amino- and carboxyl-termini of AR, respectively. *D*, PLA puncta (*red*) indicate the AR N/C interaction. PC12 parental cells, which do not express AR, included as a negative control. Scale bars, 20 μm. *E*, quantification of cells in (*C*) revealed reduced number of PLA puncta per cell in AR112Q-*versus* AR10Q-expressing cells in the presence of DHT (*top*) (*p* < 0.001), Kolmogorov-Smirnov. AR111Q F23A-expressing cells exhibited reduced puncta compared to AR112Q-expressing cells, as expected. (*p* < 0.0001), Kolmogorov-Smirnov.

As a first step to determining the mechanism underlying the protection offered by blocking the AR N/C interaction, we sought to quantify the relative levels of this interaction in cells. To do this, we used a mammalian two-hybrid system in which the DNA sequences encoding the N-terminal and C-terminal AR domains were ligated to the VP16 transactivation domain and Gal4 DNA-binding domain sequences, respectively, as previously described (12). Co-expression in HEK293T cells with a Gal4 UAS-driven luciferase reporter indicated the level of N/C interaction. As expected, DHT treatment increased the N/C interaction of both AR16Q and AR111Q, but not of AR111Q F23A, confirming that the assay reports on the N/C-interaction (Fig. 1*A*). AR111Q displayed a decreased N/C interaction compared with AR16Q (Fig. 1, *A* and *B*). To verify that this result was reflective of the N/C interaction in full-length AR, we performed a proximity ligation assay (PLA) (Fig. 1*C*) using antibodies that bind to the N- and C-termini, respectively. Quantification of PLA puncta revealed that polyglutamine-expanded AR exhibited a reduced number of PLA puncta compared with wild-type AR (Fig. 1*D*), despite slightly increased levels of polyQ-expanded AR (Fig. S1). Moreover, AR112Q bearing the F23A mutation exhibited markedly reduced PLA puncta, supporting the use of this assay to report on the AR N/C interaction. These results confirmed that polyglutamine-expanded AR exhibits a reduced N/C interaction.

### AR dimerization is increased in SBMA cell models

**Figure 2:**
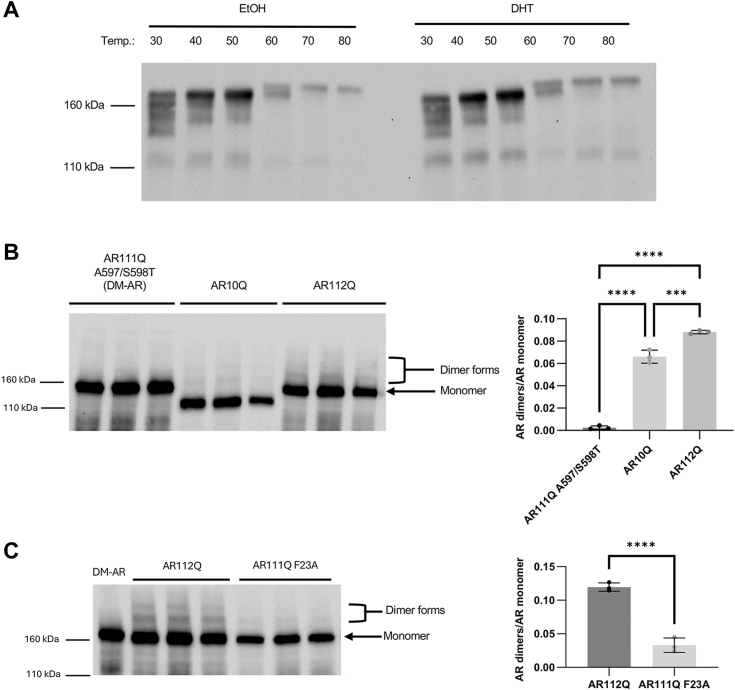
**AR dimerization is increased with polyglutamine expansion and decreased upon blocking the N/C interaction.***A*, Western blot analysis of temperature effect on AR10Q dimers. AR dimers detected *via* the dimerization blotting technique exhibit dissolution upon heating, as expected. As shown here, the proposed dimer band exhibits a wide range of molecular weights at 30 °C, which significantly condenses as heat is applied. At 60 °C, the temperature used in Figure 2, *B* and *C*, the dimer species form two distinct bands, one at the same highest molecular weight observed at lower temperatures (30–50 °C) and one at slightly higher molecular weight. The dimer band condenses further into this new highest molecular weight band at higher temperatures until it is the only detectable species at the highest incubation temperature tested. The temperature of 60 °C for further experiments (shown in Fig. 2, *B* and *C*) was selected due to it being the lowest temperature with distinct dimer bands with a wide separation between monomer and proposed dimer bands. *B*, *left*, Western blot analysis of AR dimers. Dimers are noted as two forms, the larger of which likely represents an unfolded form. Right, quantification of AR dimer forms, compared to monomer, revealed increased dimers of AR112Q compared to AR10Q. (∗∗∗*p* < 0.001, ∗∗∗∗*p* < 0.0001), one-way ANOVA with post hoc Tukey test. *C*, *left*, Western blot analysis of AR dimer forms (*left*) and quantification (*right*) reveals that blocking the AR N/C interaction reduced the ratio of AR dimers to AR monomer. (*p* < 0.0001), Student’s two-tailed *t* test.

We previously showed that the AR N/C interaction contributes to polyglutamine-dependent AR toxicity; inhibiting the interaction substantially reduced its aggregation and toxicity (12, 16). Thus, the reduced N/C interaction in AR112Q was somewhat unexpected. One possible explanation is that different forms of the AR N/C interaction, for example, intra- *versus* inter-molecular interactions, play differing roles in SBMA. We therefore investigated the state of AR dimerization using a modified native Western blotting protocol (35) that allows monomeric and multimeric AR to be resolved as distinct bands (Figs. 2, *A* and *B* and S2). The dimeric nature of the higher molecular weight bands was confirmed using a dimerization-incompetent form of AR bearing a A597T/S598T double mutation, referred to here as dimerization-mutant AR or DM AR, which was previously shown to prevent dimerization of AR (21). Increasing the temperature at which the protein lysates were prepared resulted in the apparent unfolding of the higher molecular weight species (Figs. 2*A* and S2), resulting in two bands that reflect the dimer state. Blocking AR dimerization significantly decreased the intensity of both bands (quantification in Fig. 2*B*). As shown in Figure 2*B*, polyQ-expanded AR (AR112Q) exhibited increased dimers compared to wild-type (10Q) AR. PolyQ-expanded AR dimers were decreased by the F23A mutation (Fig. 2*C*). Together, these results suggest that AR homodimerization is increased upon polyglutamine expansion and decreased by the protective F23A mutation and thus may play a role in SBMA pathology.

### Blocking AR dimerization results in decreased aggregation in PC12 cells

**Figure 3:**
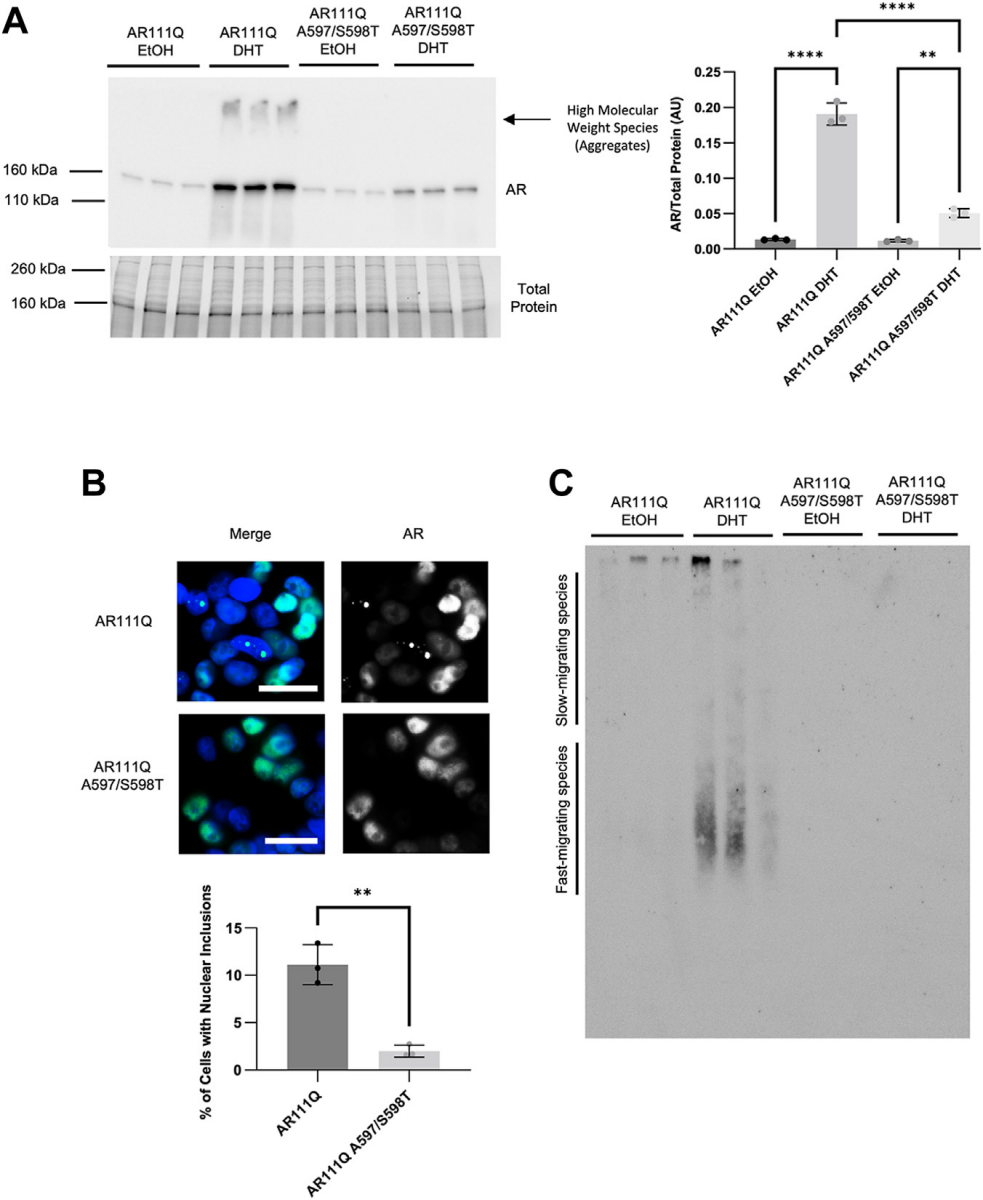
**Blocking AR dimerization reduces AR stabilization and aggregation upon DHT binding.***A*, Western blot analysis of PC12 clonal cell lines expressing AR111Q or AR111Q-DM after 6 days of treatment with DOX ± DHT revealed significantly reduced AR111Q-DM despite equivalent AR in the absence of DHT. (∗∗*p* < 0.01), one-way ANOVA with post hoc Tukey test. *B*, immunofluorescence analysis with AR-H280 antibody of AR111Q- or AR111Q A597/S598T-expressing cells treated with DOX and DHT for 6 days revealed that DM-AR formed fewer nuclear inclusions than dimerization-competent AR. (*p* < 0.01), Student’s two-tailed *t* test. Over 500 cells per coverslip were counted in triplicate. Scale bars, 20 μm. Merge, blue represents Hoechst stain. *C*, analysis of AR aggregation by SDS-AGE revealed reduced amounts of both slow-migrating and fast-migrating AR aggregation species, originally described in (37), formed by DM AR. Immunoblot with AR-H280 antibody.

To further investigate the potential role of AR dimerization in SBMA, stably transfected doxycycline (DOX)-inducible PC12 cell lines were created with polyglutamine-expanded AR (111Q) and polyglutamine-expanded DM AR (111Q A597T/S598T), both with C-terminal FLAG tags. This model has been previously shown to form nuclear inclusions, a hallmark of SBMA pathogenesis, which increase in size over time and correlate with polyQ AR toxicity (12, 36, 37). We confirmed approximately equal levels of AR in the absence of DHT (Fig. 3*A*). As expected, DHT treatment resulted in substantial stabilization of AR111Q. However, cells expressing polyQ-expanded DM AR exhibited significantly reduced DM AR stabilization in response to DHT (Fig. 3*A*). Treatment with DHT resulted in nuclear AR111Q aggregation, as expected (36, 37). Of note, DM AR formed nuclear inclusions at a reduced frequency when compared to dimerization-competent AR (Fig. 3*B*). Additionally, DM AR formed reduced levels of aggregates, detected biochemically, on both SDS-PAGE (Fig. 3*A*) and SDS-AGE (37) (Fig. 3*C*). Taken together, these data reveal that blocking AR dimerization substantially reduces its aggregation propensity.

### Blocking AR dimerization results in increased survival of infected motor neurons

**Figure 4:**
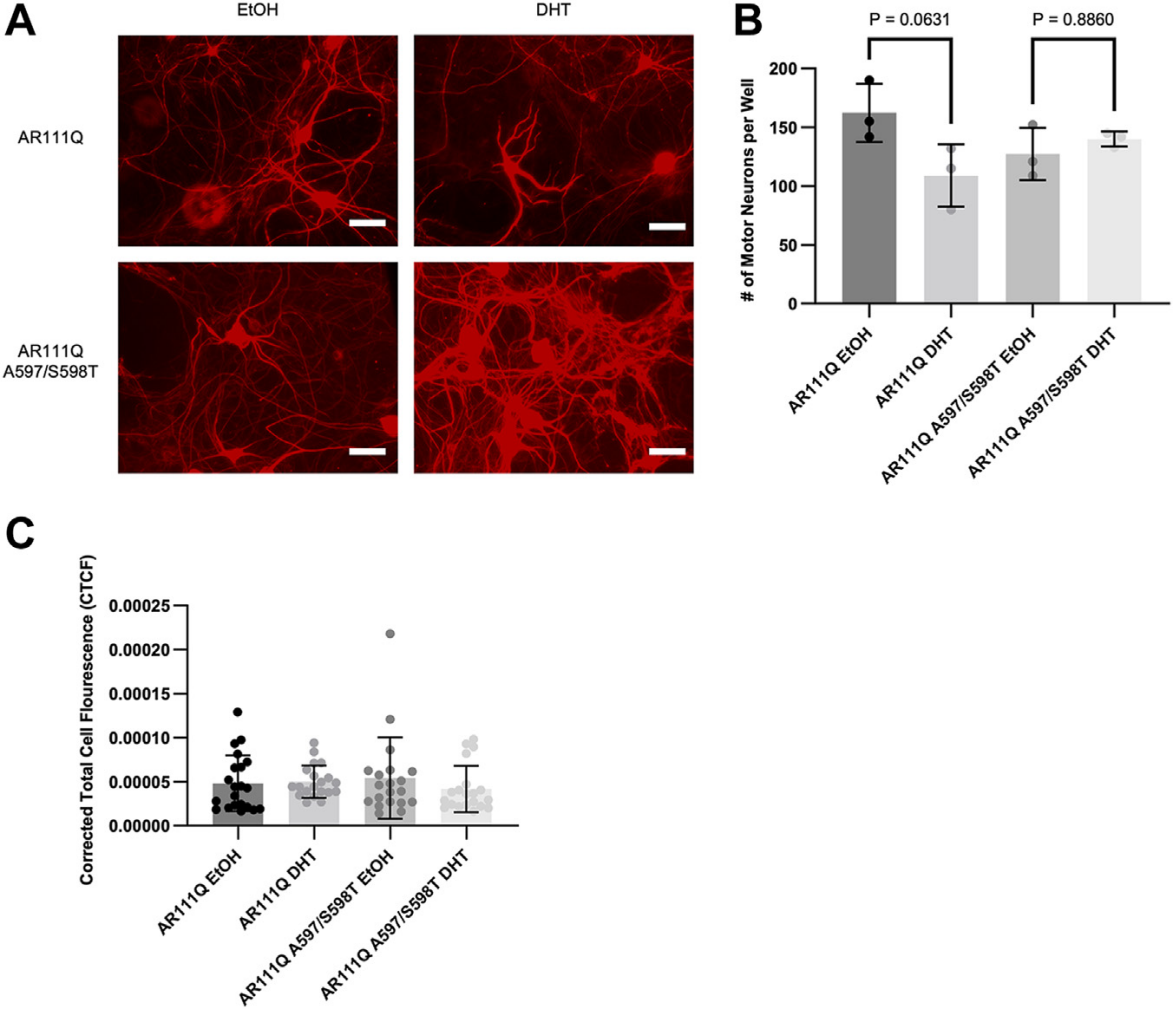
**Blocking AR dimerization protects motor neurons from DHT-induced death.***A*, dissociated spinal cord cultures were infected with AAV-AR111Q or AAV-AR111Q-A597T/S598T for 5 days, then treated for 7 days with DHT and immunostained with SMI32 antibody (uNFH). Scale bars, 100 μm. Quantification of SMI32-positive motor neurons (*B*) revealed a trending decrease in the number of motor neurons expressing AR111Q; no decrease in number of motor neurons expressing AR111Q A597/S598T was observed. 87.5% of motor neurons were infected with AAV-AR111Q and 84% of motor neurons were infected with AAV-AR111Q-A597/S598T (Fig. S3) *C*, quantification of SMI32 intensity, using corrected total cell fluorescence (CTCF), revealed no difference between dimerization-competent AR and DM-AR expressing motor neurons. One-way ANOVA with post hoc Tukey test.

We next sought to determine whether blocking AR dimerization is protective in cultured motor neurons. Primary motor neurons obtained by dissociation of embryonic mouse spinal cords were infected *via* AAV to express either dimerization-competent polyQ-AR or DM polyQ-AR. The virally delivered AR contains an exogenous nuclear localization signal to control for potential alterations in AR localization and trafficking. A high level of infectivity was observed (87.5% for dimerization-competent and 84.0% for DM AR; Fig. S3); nonetheless, DHT-dependent motor neuron death upon expressing polyglutamine-expanded AR showed only a trend towards death (*p* = 0.0631) (Fig. 4*B*), unlike our previous observations (16, 38). Expression of DM AR, on the other hand, did not result in even a trending decrease in motor neuron number (Fig. 4*B*). Visual inspection of the images revealed qualitative increases in SMI32 immunoreactivity in DHT-treated DM AR111Q-expressing motor neurons, compared to DHT-treated AR111Q-expressing motor neurons (Fig. 4*A*), an observation similar to that seen previously with neuroprotective interventions (6, 16, 39, 40). Quantification of SMI32 immunoreactivity in motor neuron somas did not, however, reveal a quantitative difference (Fig. 4*C*), suggesting that the increased intensity may result from enhanced levels of neurofilament heavy chain (NFH) in motor axons. Quantification to assess this question was complicated by the hetergeneous nature of the cultures, however. Nonetheless, the lack of even a trending decrease in motor neuron number upon DHT treatment of DM AR-expressing motor neurons suggests that blocking AR dimerization may potentially be protective in SBMA.

### Blocking AR dimerization results in altered subcellular localization of AR

**Figure 5:**
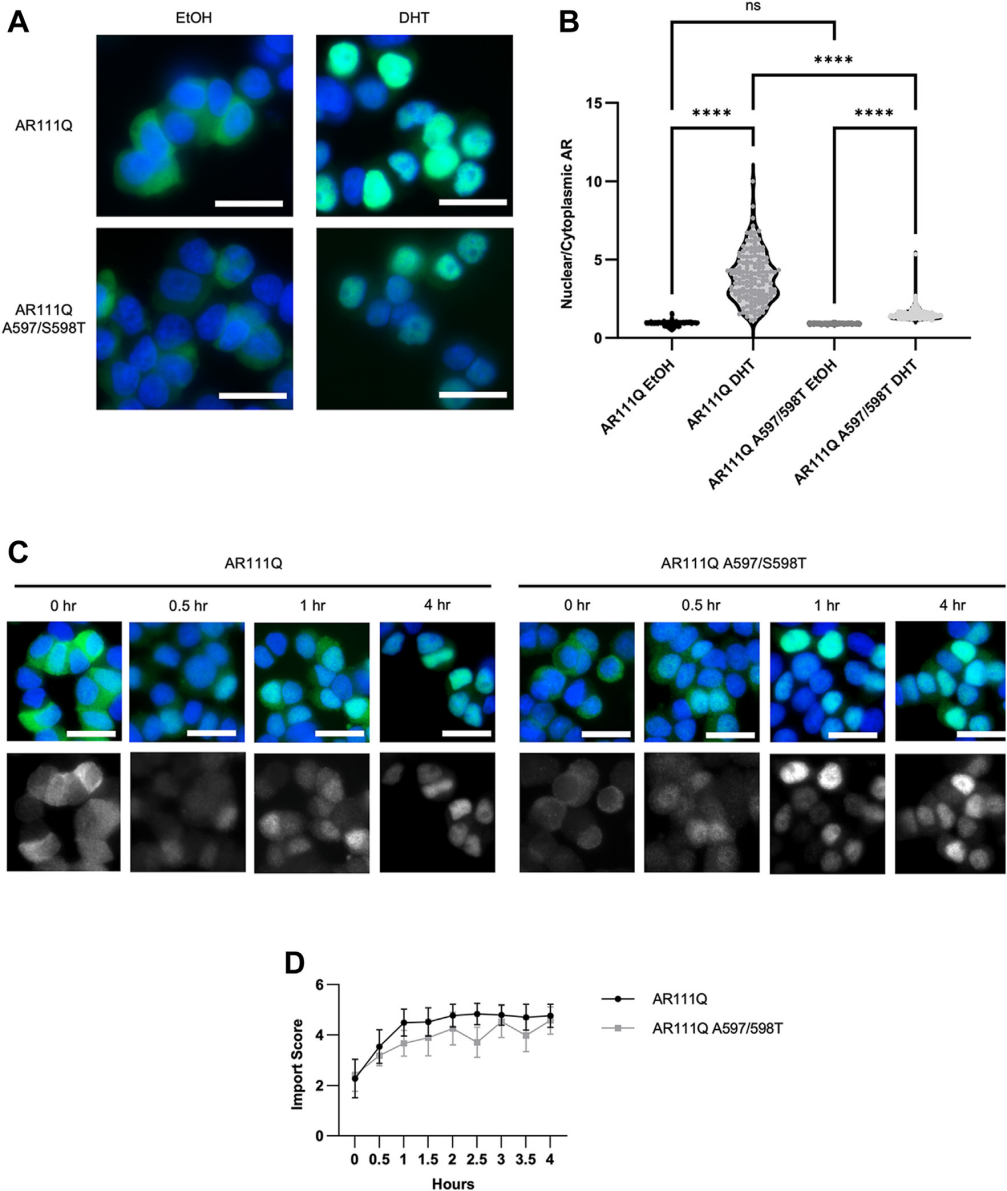
**Blocking AR dimerization leads to an altered nuclear-cytoplasmic ratio without differences in AR nuclear import.***A*, immunofluorescence analysis of AR localization revealed that DM AR exhibited similar subcellular localization to dimerization-competent AR in the absence of DHT but was more cytoplasmic in the presence of DHT. Scale bars, 20 μm. *B*, quantification of images as in (*A*) in over 150 cells per coverslip in triplicate. (∗∗∗∗*p* < 0.0001), one-way ANOVA with post hoc Tukey test. *C*, immunofluorescence analysis of AR localization over time following DHT addition revealed no change in DM-AR nuclear import. Scale bars, 20 μm. *D*, quantification of images in (*C*) for over 170 cells per coverslip in triplicate. Images in the top row of (*C*) are a merge of Hoechst nuclear stain (*blue*) and AR (*green*).

Given the results suggesting a role for dimerization in polyQ-expanded AR aggregation and toxicity, we sought to investigate the potential mechanism underlying these results. We observed slightly more cytoplasmic AR in DM AR-expressing cells in the presence of DHT (Figs. 3*B* and 5A), compared to dimerization-competent AR. In the absence of DHT, AR subcellular localization was unaffected by the dimerization mutation (Fig. 5, *A* and *B*). However, following DHT treatment, a larger fraction of DM AR was retained in the cytoplasm compared to that observed in dimerization-competent AR (Fig. 5*B*). This difference persisted over time, from two to 6 days of DHT treatment (Fig. S4). We and others previously showed that differences in AR localization and trafficking play a role in polyQ-AR toxicity (38, 41, 42), leading us to further investigate AR trafficking in these cells. Given the proximity of the dimerization domain to the AR nuclear import sequence (43), we started our investigation by examining AR nuclear import. Quantification of AR localization following DHT treatment revealed no significant difference in AR nuclear import over the course of 4 h (Fig. 5, *C* and *D*). We also evaluated AR nuclear export using a heterokaryon assay, which revealed no difference in the export of dimerization-competent AR *versus* DM AR (Fig. S5). These results indicate that the observed altered localization of DM AR does not result from altered nuclear import or export.

### Blocking AR dimerization results in altered AR degradation

**Figure 6:**
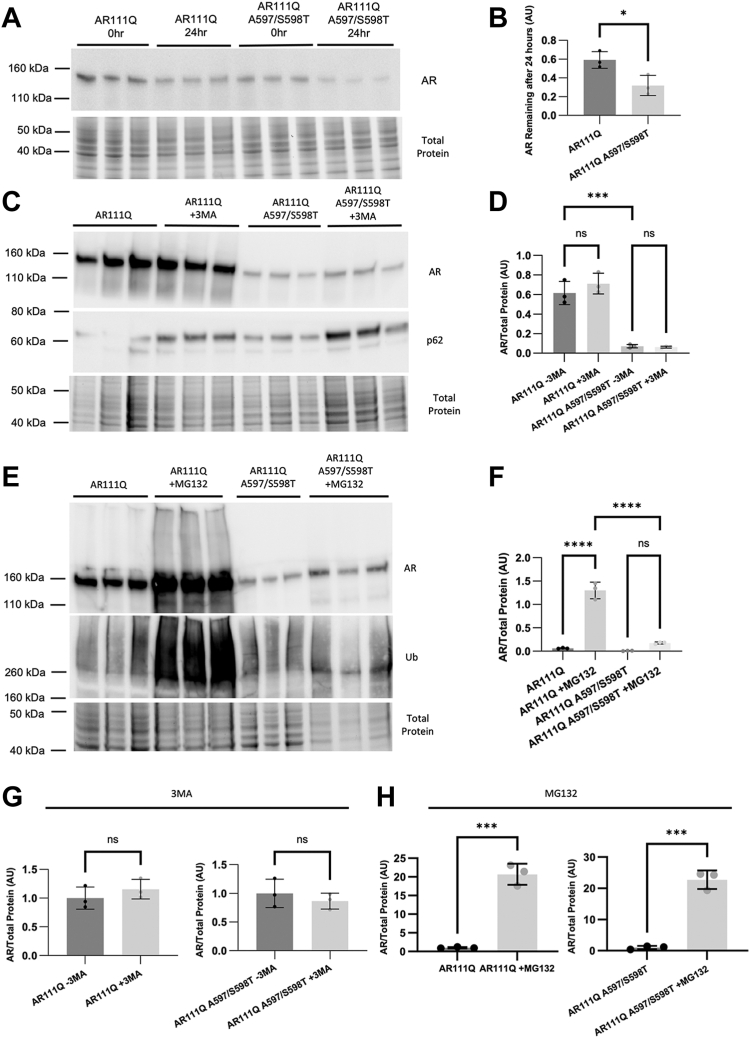
**Blocking AR dimerization results in increased androgen receptor degradation.***A*, Western blot analysis of AR111Q and AR111Q-DM following Dox washout and cycloheximide treatment revealed enhanced turnover of AR111Q-DM. *B*, analysis of AR remaining after 24 h revealed that AR111Q-DM was degraded nearly two-fold more than AR111Q in 24 h (∗*p* < 0.05), Student’s two-tailed *t* test. *C*, inhibition of autophagy with 3-MA did not restore levels of DM AR. *D*, quantification of western blots in (*C*) (∗∗∗*p* < 0.001), one-way ANOVA with post hoc Tukey test. *E*, treatment with the proteasome inhibitor MG132 increased the levels of both dimerization-competent and DM-AR. *F*, quantification of western blots in (*E*) (∗∗∗∗*p* < 0.0001), one-way ANOVA with post hoc Tukey test. *G*, quantification of western blots in (*C*) with data normalized to AR/total protein without 3-MA. Student’s two-tailed *t* test. *H*, quantification of Western blots in (*E*), normalized to AR/total protein without MG132. (∗∗∗*p* < 0.001), Student’s two-tailed *t* test.

The altered localization of DM AR might alternatively be explained by altered AR turnover in the presence of DHT, particularly given the reduced DHT-induced stabilization of DM AR seen in Figure 3*A*. Quantification of AR protein stability following 24 h of cycloheximide treatment revealed enhanced turnover of DM polyQ-AR (Fig. 6, *A* and *B*). Treatment with the autophagy inhibitor 3MA resulted in only a small increase in AR levels in both cell lines (Fig. 6, *C* and *D*). Inhibition of the proteasome with MG132 resulted in a more substantial increase in AR levels of DM AR, although not to the level of dimerization-competent AR (Fig. 6, *E* and *F*). Nonetheless, the impact of proteasome inhibition on AR levels in each cell line, relative to AR levels in the absence of proteasome inhibition, revealed an equivalent robust increase in AR protein (Fig. 6*H*), unlike that observed upon autophagy inhibition (Fig. 6*G*). The difference in AR stabilization upon proteasome and autophagy inhibition is consistent with previous literature as AR is known to be primarily degraded by the proteasome (41). However, the inability of either treatment to restore levels of DM AR to that of dimerization-competent AR was somewhat surprising. Nonetheless, the reduced half-life of DM AR likely explains its reduced aggregation and toxicity and indicates that AR dimerization represents an additional step in AR metabolism that contributes to SBMA pathology.

## Discussion

This work identifies a significant role for AR homodimerization in SBMA. We found that polyglutamine-expanded AR exhibited increased dimerization compared with wild-type AR. Moreover, blocking the N/C-interaction, a mutation shown to be protective (12, 16) resulted in a decrease in dimerization. Additionally, blocking AR dimerization through genetic mutation of the D-box within the second zinc finger resulted in decreased AR aggregation in PC12 cells. These effects were correlated with increased cytoplasmic localization of AR, which has also been previously shown to be protective in SBMA (38, 41), and increased AR degradation. These novel findings uncover a new mechanism, AR dimerization, by which polyQ-expanded AR may lead to toxicity in SBMA as well as a potential new avenue for preventing or reversing polyQ-expanded AR toxicity.

The initial finding presented here, that polyglutamine-expanded AR showed decreased N/C interaction, was somewhat surprising. We expected the N/C interaction to be increased in polyglutamine-expanded AR since blocking this interaction is protective, in *in vitro* and *in vivo* models of SBMA (12, 16). Other studies, however, have also found that polyglutamine expansion significantly alters the structure of the N-terminus of AR, which may explain the alteration of the N/C interaction observed here (44).

Our observation that DHT-dependent cell death of motor neurons expressing dimerization-competent AR112Q was only statistically trending, albeit substantial (Fig. 4), prevented our ability to determine whether blocking dimerization is protective in SBMA. Nonetheless, the lack of even a trending decrease in cell number upon blockade of AR homodimerization suggests that prevention of AR dimerization may be protective. While the PC12 cell model is often used to assess toxicity, the PC12 cell lines described here express low levels of AR, insufficient to allow the assessment of cell death. Regardless, the significant effects of blocking AR dimerization on the aggregation, subcellular localization, and stabilization of AR provide both novel and promising support to the potential benefits of blocking AR homodimerization in SBMA.

This work has uncovered an additional unexpected result, the incomplete rescue of DM AR levels after proteasome blockade. Previous work has identified the ubiquitin-proteasome pathway as the main method by which AR is degraded and that this degradation is tied to the nuclear export of AR (41). In that study, adding a CRM1-mediated nuclear export signal was able to more substantially increase AR degradation by the proteasome over the same 16-h timepoint used here (41). However, in the present study, no increase in AR nuclear export was observed upon mutation of the AR to prevent its dimerization. Combined with the relatively long half-life of AR in cells, it is possible that we do not see a complete rescue of DM AR levels after inhibiting the proteasome due to distinct trafficking-dependent regulation of AR turnover in these examples. Nonetheless, it remains likely, based on our results, that proteasomal degradation is the cause of the decreased hormone-induced stabilization we see in DM AR. Further study of the mechanism by which this increased degradation occurs, including evaluation of post-translational modifications and interactions with proteins (*e.g.*, ubiquitin E3 ligases, deubiquitinases) that regulate AR proteasomal degradation, is of significant interest and should provide more insight into the mechanism by which preventing AR dimerization changes its intracellular stability.

The increased localization of DM AR to the cytoplasm of cells is intriguing. Given the lack of significant difference in either AR nuclear import or export, it may be that a portion of AR remains in the cytoplasm of cells and is simply never imported to the nucleus. Localization of AR to another subcellular location may contribute to this observation. AR has been previously observed in several subcellular locations, including mitochondria (45, 46, 47, 48), which may be mediated by a proposed mitochondrial localization sequence in the first 36 amino acids of the AR (45). Metabolic changes and alterations of mitochondria have also been described in several SBMA models, lending support to the potential role of mitochondria-associated AR being important in SBMA pathogenesis (49, 50, 51, 52, 53). However, more work is needed to understand the mechanism behind the observed increased cytoplasmic localization of DM AR as well as the significance of this localization to its protective effects.

Although this study originally sought to better understand the mechanism by which blocking the N/C interaction leads to protection in SBMA models, the changes in cellular localization and degradation seen in DM AR are distinct from those seen when the N/C interaction is blocked (12). It is possible, therefore, that multiple mechanisms contribute to the neuroprotection conferred by blocking the AR N/C interaction.

The effects of blocking AR homodimerization that we observed here invite the question of whether changes in AR binding to DNA contribute to these effects. Although the DNA binding of DM AR has not been measured, it likely exhibits decreased DNA binding due to its inability to bind to the palindromic, dihexameric repeat sites known as AREs; this process stabilizes AR dimers (26, 54, 55, 56). However, AR that is unable to bind to DNA is still able to dimerize (21). This suggests that, even if DM AR exhibits reduced DNA binding, this feature likely does not underlie the effects of blocking AR dimerization that we describe in our study. Nonetheless, while outside the scope of this study, additional investigation into the role of DNA binding in dimerization-dependent AR stabilization may provide further mechanistic insights into this process.

Our novel discovery described here on the important role of dimerization in SBMA may have an important impact on the search for an effective treatment for the disease. The dimerization domain contains a binding pocket that is considered “druggable.” Indeed, several compounds have been identified that bind to this region of AR and block homodimerization (57, 58, 59). These may present an opportunity to target this newly identified mechanism and present promising candidates for potential development as therapeutics for SBMA. Additionally, it is possible that the involvement of the ligand-binding domain in homodimerization could play a role in the mechanism identified here, although our results reveal a substantial effect upon mutation of only the D-box dimerization motif. There exists a binding pocket within the LBD homodimerization domain, for which several small molecules have been designed (27, 28, 30, 60, 61). Although more research is needed to determine if this domain results in a similar effect as the A597/S598T double mutation, it may present an additional method to target this mechanism for therapeutic effect in SBMA.

## Experimental procedures

### Generation of cell lines

The dimerization-deficient AR plasmid was made using site-directed mutagenesis (QuikChange II XL Mutagenesis kit, Agilent technologies) to mutate A597 to T597 and S598 to T598 in a Tet-On pTRE-AR plasmid (36). 3xFLAG sequence (3× DYKDDDDK) was attached in the frame to the 3′ end of the AR cDNA *via* standard cloning. These mutations as well as the CAG length were sequence-verified.

Stable transfection of plasmids was achieved by co-transfection (Lipofectamine 2000) with a plasmid conferring hygromycin resistance (pTK-Hygro) in a 4:1 ratio into Tet activator-expressing PC12 cells. Stable transformants were selected with 200 μg/ml hygromycin. Clonal lines were screened for AR expression level and polyQ length using Western blot analysis (AR-H280; Santa Cruz). Clones expressing a single AR species at the desired level were then subjected to genomic DNA collection (DNA-Easy Blood & Tissue kit, Qiagen) and sequence analysis. The concentration of doxycycline needed to induce equal amounts of AR in the absence of hormone was determined using Western blot analysis.

### Cell culture and reagents

Tet-on pheochromocytoma-derived rat PC12 cells expressing the indicated AR genotype were maintained in Dulbecco’s modified Eagle’s medium (DMEM) supplemented with 10% heat-inactivated horse serum, 5% heat-inactivated fetal bovine serum, 2 mM L-glutamine, 100 U/ml penicillin/streptomycin, 200 μg/ml hygromycin and 100 μg/ml G418 sulfate. Cells were maintained at 37 °C and 10% CO_2_. All experiments were performed with charcoal-stripped media to remove serum-derived androgens. AR activation was induced by treatment with 10 nM DHT (Sigma Aldrich).

### Mammalian-2-hybrid assay

HEK293 cells were transfected with pVP16-AR111Q (amino acids 12–660) and pM-Gal4DBD-AR (amino acids 624–919) in a 1:1 ratio as well as pG5-Luc (2.5:1 ratio to pVP16) and pRL-TK (1:20 ratio to pVP16) using Lipofectamine 2000 (Invitrogen) following the manufacturer’s instructions. Cells were allowed to incubate for 48 h in the presence or absence of 10 nM DHT before collection using the Dual-Luciferase Reporter Assay Kit (Promega) as per the manufacturer’s instructions. Luminescence was measured using a plate reader (Tecan). Firefly luciferase signal was normalized to the Renilla luciferase signal.

### Proximity ligation assay

Cells were plated on poly-D-lysine-coated coverslips and treated with doxycycline with or without 10 nM DHT for 24 h. Cells were fixed with 4% paraformaldehyde. Duolink PLA fluorescence protocol was followed as per the manufacturer’s instructions (DUO92008, Sigma-Aldrich) with primary anti-AR antibodies ARC19 (Santa Cruz Biotechnologies) and AR318 (Leica Biosystems). Nuclei were stained with Hoechst before imaging. All images were acquired using the same exposure on a Leica DMR Fluorescence Microscope (Leica Microsystems GmbH, Wetzlar) and ProgRes software. The number of puncta was determined in at least 100 cells per condition. The experiments were repeated three times. The experimenter was blinded to experimental conditions.

### Immunoblotting

Cells were treated for 48 h with doxycycline and 10 nM DHT before collection in 2% SDS lysis buffer (2% SDS, 10 mM Tris, 150 mM NaCl, 0.5 mg/ml PMSF, Roche cOmplete Tablets protease inhibitors). Lysates were sonicated using four 1-s pulses with a probe sonicator (QSonica) and protein levels were assessed by DC total protein concentration assay (Bio-Rad). Equal protein amounts in Laemmli buffer were boiled and electrophoresed on SDS-PAGE using 10% stain-free agarose gels (Bio-Rad). Gels were imaged before transfer on a ChemiDoc MP (Bio-Rad) in order to obtain total protein signal. Proteins were the transferred to 0.45 um PVDF membrane (Immobilon-P), incubated in 5% milk in TBST (10 mM Tris-HCl, pH 8.0, 150 mM NaCl, 0.1% Tween-20) for 60 min at room temperature followed by incubation with primary antibodies overnight at 4 °C. Primary antibodies used include anti-AR (H280; Santa Cruz), anti-SQSTM1 (#5144; Cell Signaling), and anti-ubiquitin (P4D1; Santa Cruz Biotechnologies). Following incubation with HRP-conjugated secondary antibodies, detection was performed using ECL substrate (Bio-Rad) followed by imaging on ChemiDoc MP.

### Immunoblotting for AR dimers

Cells were treated for 24 h with doxycycline followed by doxycycline washout and treatment for a further 24 h with 10 nM DHT. Cells were collected in 0.5% SDS extraction buffer (0.5% SDS, 0.1% Triton X-100, 10 mM HEPES, 1 mM EDTA) (derived from (62)) and sonicated on ice until no longer viscous. Lysates were then incubated at 60 °C or the indicated temperature for 30 min to allow for homogenization. Non-denaturing Laemmli buffer containing neither SDS nor beta-mercaptoethanol was added before samples were electrophoresed on 10% SDS-PAGE. Proteins were transferred for immunoblotting as described above. Samples were always electrophoresed on the day of collection to avoid freeze-thaw cycles. This protocol was derived from that in (35).

### Immunostaining

Cells were cultured on poly-D-lysine-coated coverslips, then fixed with 4% paraformaldehyde in PBS for 30 min. Following permeabilization and blocking in 0.3% Triton X-100 and 5% normal donkey serum (NDS), cells were incubated overnight at 4 °C with ARH280 (1:100) in 2% NDS in PBS. Cells were washed in PBS and incubated at room temperature for 1 h with fluorescent-conjugated secondary antibodies. Cells were stained with Hoechst (1:500), mounted using Vectashield (Vector Laboratories), and imaged using either Leica DMR Fluorescence Microscope (Leica Microsystems GmbH) or EVOS M7000 Microscope (ThermoFisher Scientific). The percentage of cells with nuclear inclusions was evaluated by staining cells from various experimental conditions with anti-AR antibody H280. Five hundred cells were quantified per coverslip with three coverslips per experimental condition. Each experiment was repeated three times. The experimenter was blinded to experimental conditions.

### SDS-agarose gel electrophoresis

Cells were treated in the same protocol as for immunoblotting and collected in 2% SDS lysis buffer (2% SDS, 10 mM Tris, 150 mM NaCl, 0.5 mg/ml PMSF, Roche cOmplete Tablets protease inhibitors). Samples were sonicated and protein concentration was measured *via* DC total protein concentration assay (BioRad). Sample volumes were adjusted such that equal amounts of protein (200 μg) were loaded in each lane. Gels were prepared by boiling 1% (w/v) agarose in 375 mM Tris-HCl pH 8.8 before the addition of 0.1% SDS (v/v). Samples were then diluted with Laemmle buffer without beta-mercaptoethanol followed by boiling at 90 °C for 5 min. Samples were then loaded on the gel and electrophoresed for 16 h at 55 V at 4 °C. Protein transfer was performed using an Owl HEP semi-dry blotting unit at 20 V for 3 h. Membranes were developed as usual for a Western blot.

### Dissociated spinal cord culture

The studies as described were approved by the Institutional Care and Use Committee of Thomas Jefferson University. Dissociated mouse spinal cord cultures were established as previously described (63). Briefly, embryonic mouse spinal cords were dissected from embryonic day 13.5 C57Bl6/J mice. Spinal cords were dissociated with trypsin and plated on poly-D-lysine-coated 96-well plates. Cultures were grown in glia-conditioned media containing minimal essential medium, 3% charcoal-stripped horse serum, 35 mM NaHCO_3_, 0.5% dextrose, 1% N3, and 10 nM 2.5S nerve growth factor for 3 weeks. Cultures were then infected with AAV1 to express AR111Q or AR111Q A597/S598T for 5 days, followed by treatment with DHT or ethanol (vehicle) for another 7 days. Infectivity was >80% as assessed by staining with anti-AR (H280) antibody. Motor neurons were identified by the presence of unphosphorylated neurofilament heavy chain (SMI32 immunoreactivity) and morphology. The experiment was performed three times and at least three wells per condition were analyzed in each experiment. The experimenter was blinded to experimental conditions during the analysis of images. SMI32 expression was quantified by measuring the corrected total cell fluorescence (CTCF) (CTCF = Integrated grey density – (Area of selected cell ∗ Mean fluorescence of background readings)) of the cell body of 20 motor neurons per condition. The fluorescent intensity of each well was quantified by measuring the integrated grey density of the image of each well comprised of individual views stitched together into one file.

### AR import assay

To assess AR import, PC12 cells expressing either dimerization-competent or -incompetent AR were plated on poly-D-lysine coated coverslips and treated with doxycycline for 24 h. Cells were then washed, treated with 10 nM DHT for 0 to 4 h and immunostained with anti-AR (H280) as described above. All images were acquired using the same exposure on Leica DMR Fluorescence Microscope (Leica Microsystems GmbH) and ProgRes software. Images were acquired with the same exposure time. 50 cells per coverslip were analyzed with three coverslips per treatment condition. Analysis was performed with ImageJ by selecting three random but equal areas of either the nucleus or cytoplasm. The mean gray value in each area was averaged across all three, and then this average for the cytoplasmic compartment was divided by the average for the nuclear compartment. The experimenter was blinded to experimental conditions during both the imaging and analysis portion of this experiment. This experiment was repeated three times.

### AR stabilization assays

PC12 cells expressing either dimerization-competent or -incompetent AR were treated with doxycycline for 48 h to induce AR expression. At this point, cells were either washed and lysed (0-h washout) or treated with 10 nM DHT and 50 μg/ml cycloheximide for an additional 24 h. Cell lysates were resolved *via* SDS-PAGE as described above. AR signal was quantified relative to the total protein signal. Each experiment was performed in triplicate and the experiment was repeated 3 times.

The method of AR degradation was assessed by treating PC12 cells with either dimerization-competent or -incompetent AR with doxycycline for 24 h followed by washout and further treatment with either MG132 (20 μM, ThermoFisher Scientific) or 3MA (5 mM, Selleck Chem) for 16 h. Cells were then lysed and proteins resolved *via* SDS-PAGE as described above. AR signal was quantified relative to the total protein signal. Each experiment was performed in triplicate and the experiment was repeated 3 times.

### Heterokaryon shuttling assay

The heterokaryon shuttling assay was carried out as previously described (41). PC12 and NIH/3T3 cells were plated and incubated for 24 h in charcoal-stripped serum-containing media. PC12 cells were then treated with doxycycline for 24 h to induce the expression of AR and then with 10 nM DHT and 10 μg/ml cycloheximide for 2 h. Concurrently, NIH/3T3 cells are incubated in two uM CellTracker Orange (ThermoFisher) for 45 min in serum-free media followed by incubation in complete media for 1 h. All cells were then trypsinized, combined in a 1:1 ratio, then pelleted and resuspended in 100 uL 50% PEG-1500 (w/v) and allowed to incubate for 2 min at room temperature. Samples were then diluted with serum-free media to halt fusion and plated onto poly-D-lysine coated coverslips. Cells were cultured in complete media supplemented with 10 nM DHT and 10 μg/ml cycloheximide for 4 h at 37 °C, then fixed with 4% paraformaldehyde and immunostained as described above.

### Statistical analysis

In the case of multiple comparisons, statistical significance was determined by one-way ANOVA with post hoc Tukey test. Comparisons between only two experimental conditions were carried out using Student’s one-tailed *t* test unless otherwise indicated. The comparison of distributions was measured with the Kolmogorov-Smirnov test. A *p* value less than 0.05 was considered significant. Data were analyzed using Prism 10.

## Data availability

Additional data is available upon request. (Diane Merry, corresponding author)

## Supporting information

This article contains supporting information (41).

## Conflict of interest

The authors declare that they have no conflicts of interest with the contents of this article.
