# Supplementary material for: Blocking the dimerization of polyglutamine-expanded androgen receptor protects cells from DHT-induced toxicity by increasing AR turnover
Source: J Biol Chem. 2024 Mar 29;300(5):107246. doi: 10.1016/j.jbc.2024.107246 (PMC11067348; doi:10.1016/j.jbc.2024.107246)
Supplement: Supporting Figures S1–S5 [file mmc1.pdf]

Supporting Information

Blocking the dimerization of polyglutamine-expanded androgen receptor protects cells from DHT-induced toxicity by increasing AR turnover

Allison Lisberg [1], Yuhong Liu[1], and Diane E. Merry[1]*

[1]Department of Biochemistry and Molecular Biology, Thomas Jefferson University, Sidney

Kimmel Medical College, Philadelphia, PA 19107

Included:

Figure S1: Cell lines used in the PLA in Figure 1C show approximately equal AR expression by Western blot analysis.

Figure S2: AR multimers detected via the dimerization blotting technique exhibit dissolution upon heating, as expected. This figure is a further clarification of Fig. 2A.

Figure S3: AR immunofluorescence in AAV1-infected neurons revealed significant expression in a majority of motor neurons.

Figure S4: The alteration in AR cellular localization in AR111Q A597/S598T after DHT treatment is observed at multiple time points.

Figure S5: Blocking AR dimerization does not change AR nuclear export.

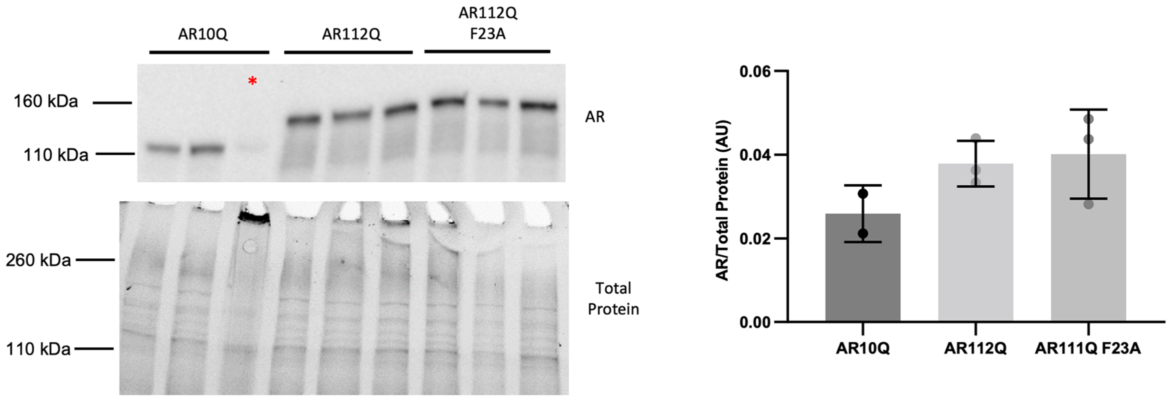

Figure S1: Cell lines used in the PLA in Figure 1C show approximately equal AR expression by Western blot analysis. Lane 3 (marked with an asterisk) ran poorly, as indicated by the protein staining stuck in the well of the total protein blot, and thus was not included in the quantification. There are no significant differences in the expression levels of these lines, as tested by a one-way ANOVA. Though AR112Q does show a slightly increased average expression level, this further highlights the impact of the reduced PLA signal seen in Figure 1C and D.

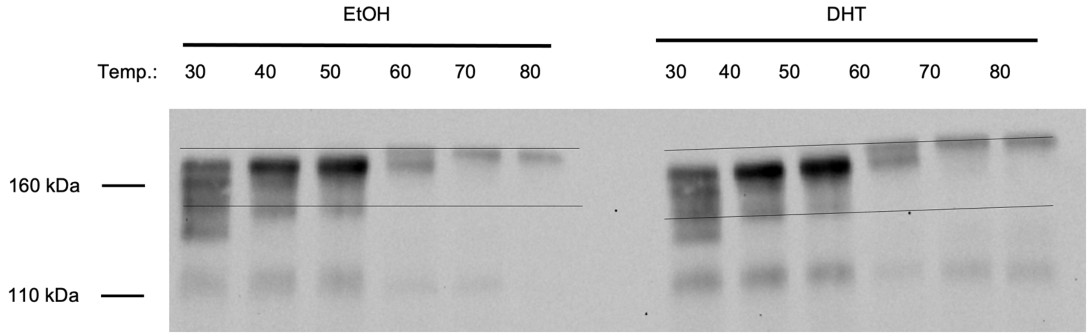

Figure S2: Image from Fig. 2A, showing AR10Q multimers detected via the dimerization blotting technique. Multimers exhibit dissolution upon heating, as expected. Lines parallel to the monomeric species- the lowest molecular weight band- have been added to better track the heat unfolding of these species. It is also notable that, in addition to heat unfolding, we also see loss of AR at higher temperatures, potentially decreasing the observed dimeric species. However, this heat-induced degradation also impacts the monomeric species and would thus be cancelled out in the quantification of these assays.

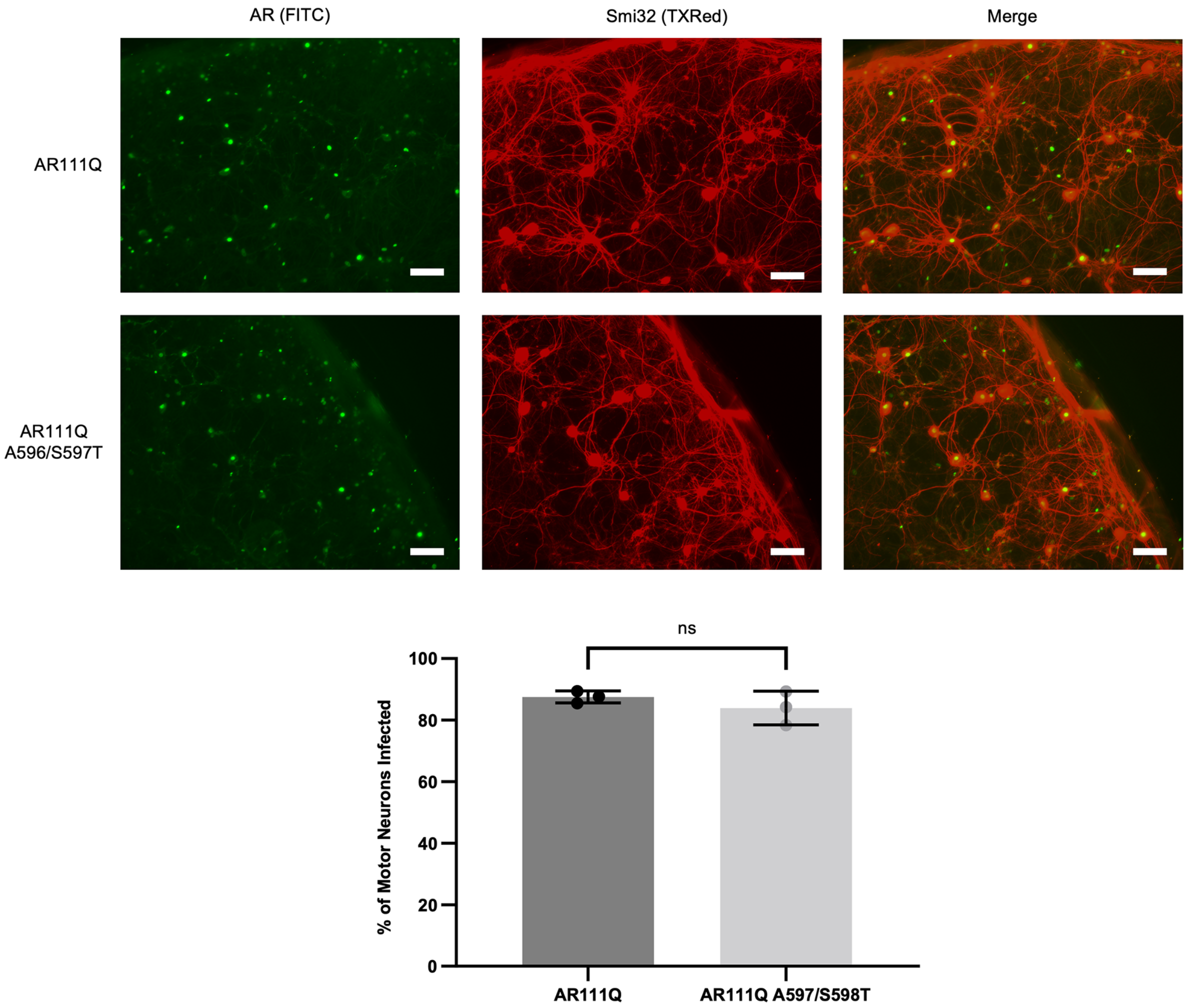

Figure S3: AR immunofluorescence in AAV1-infected neurons revealed significant expression in a majority of motor neurons. AR (green) nuclei in motor neurons (red), as identified by visual distinction using morphologic characteristics, were compared to motor neurons without AR-stained nuclei. Scale bars, 200 μm. As pictured and quantified, there was a high rate of infectivity of both AR constructs into motor neuron culture. Additionally, the infectivity rate is similar for both viruses, simplifying analysis of these experiments. Quantification of infectivity of these viruses was compared via Student's two-tailed t-test and was determined to be nonsignificant.

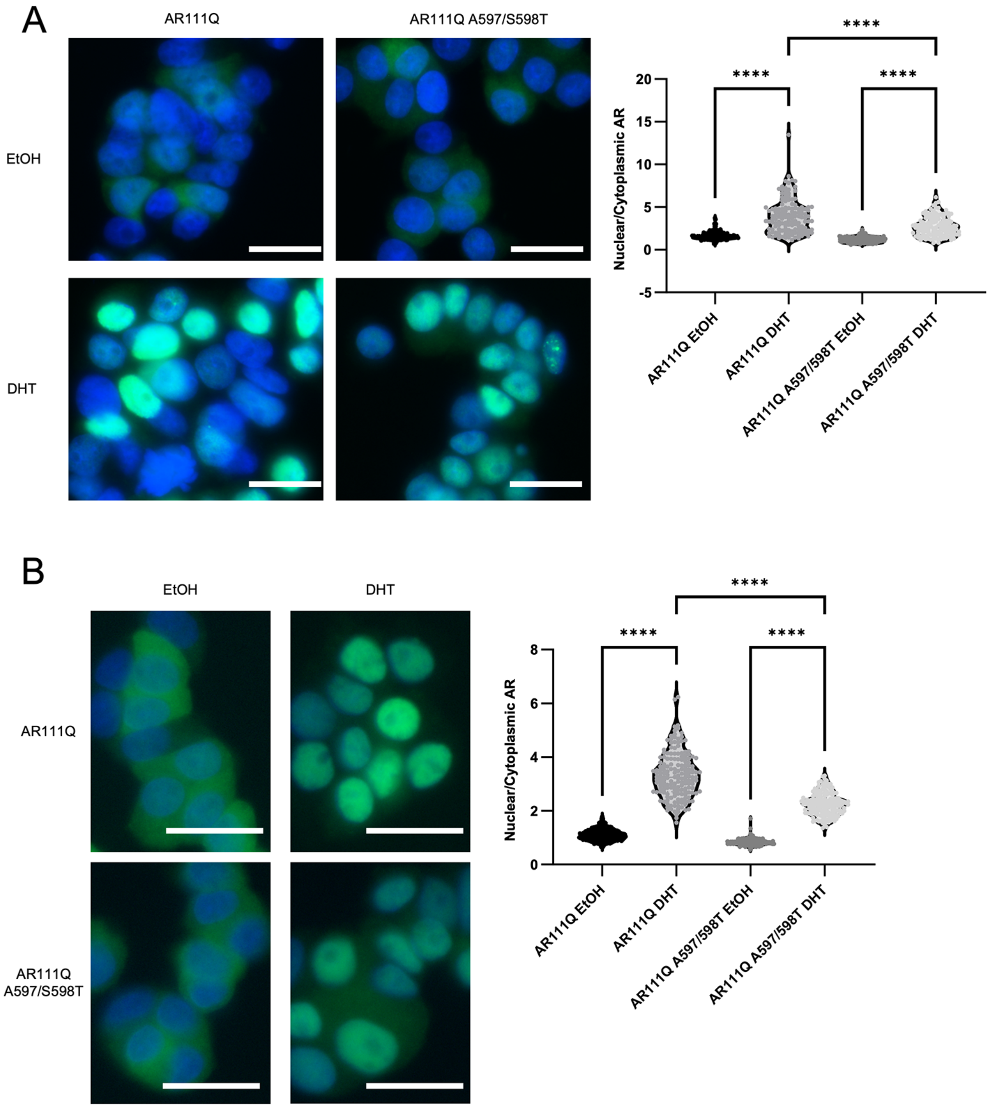

Fig. S4: The alteration in AR cellular localization in AR111Q A597/S598T after DHT treatment is observed at multiple time points. Although the cytoplasmic AR to nuclear AR ratio remained the same in the absence of DHT, nuclear AR was decreased in AR111Q A597/S598T expressing cells after both 2 days of DHT treatment (A) and 3 days of DHT treatment (B). In all images, blue represents Hoechst nuclear staining and green represents AR staining. Scale bars, 20 μm. (**** $p<0.0001$), one-way ANOVA with post hoc Tukey test. Over 150 cells per coverslip were quantified in triplicate.

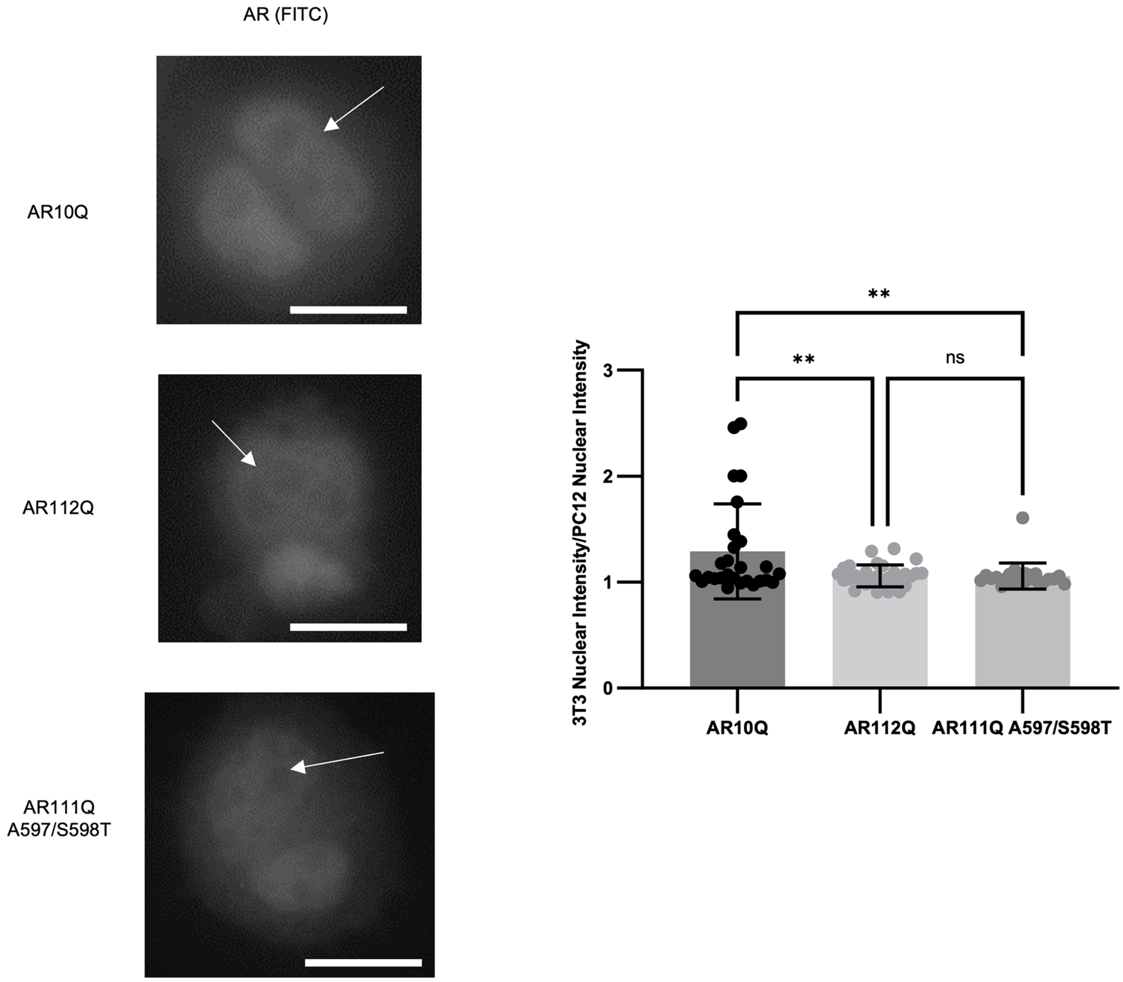

Fig. S5: Blocking AR dimerization does not change AR nuclear export. AR nuclear export, as determined through a heterokaryon-based assay, was reduced for polyglutamine-expanded AR, as shown previously (41). Blocking AR dimerization did not increase this export. (** $p<0.01$), one-way ANOVA with post hoc Tukey test. Scale bars, 20 μm.
